# A clinical inventory of moderators of tic severity in Tourette's Syndrome

**DOI:** 10.1192/bjo.2021.656

**Published:** 2021-06-18

**Authors:** Francesca Conti, Himanshu Tyagi

**Affiliations:** 1 UCL Division of Psychiatry; 2 UCL Queen Square Institute of Neurology, The National Hospital for Neurology and Neurosurgery

## Abstract

**Aims:**

Changes in the severity of tics in Tourette's syndrome (TS), as seen with variations in the intensity or frequency of tics, can be moderated by a variety of independent factors such as external or internal stimuli. Identifying such moderators has important clinical implications as it can aid clinicians in adjusting interventions. Hence, based on our previous review of tic-severity moderators, we developed a clinical inventory of moderating variables for motor and vocal tics for inclusion in the new version of the Queen Square Proforma for Tourette's Syndrome to aid initial assessments in the National Tourette Syndrome Service's Outpatient Clinic for Adults.

**Method:**

A review of tic-severity moderators was previously carried out by the authors to investigate the kinds of moderators and their worsening, improving or neutral effects on tic severity. Based on this a semantic thematic analysis of the identified moderators was carried out and themes were developed based on appropriate and relevant MeSH terms in order to create the categories and items of the clinical inventory.

**Result:**

Seventy-three different tic severity moderators were identified, the most common being exercise, sleep, peer victimisation, psychosocial stress, watching TV, academic activities and distraction. Twenty-nine themes emerged from the thematic analysis which were then used to update the clinical inventory of tic severity moderators. The review also highlighted the subjectivity of these moderators’ effects on tic severity as some moderators were tic-worsening in some individuals and tic-improving or neutral in others, which is contrary to the current dichotomous understanding.

**Conclusion:**

The updated clinical inventory of tic severity moderators invites researchers and clinicians to be more aware of the existence, variability and subjective effects of these tic severity moderators in individuals with TS, as these have been previously looked in a dichotomous way. By better identifying tic-severity moderators and their worsening, improving or neutral effects on tic severity this clinician rated inventory will have potentially important, direct implications for the management and treatment of tics.

